# Mild intrahepatic cholestasis of pregnancy—A call to redefine diagnostic criteria and consider a new management paradigm

**DOI:** 10.1002/pmf2.70245

**Published:** 2026-01-21

**Authors:** Minhazur Sarker, Rachel Wiley, Timothy Wen, Leah M. Lamale-Smith, E. Nicole Teal, Ukachi Emeruwa, Chia-Ling Nhan-Chang, Gladys A. Ramos, Cynthia Gyamfi-Bannerman, Jerasimos Ballas

**Affiliations:** 1 Division of Maternal-Fetal Medicine, Department of Obstetrics, Gynecology and Reproductive Science, University of California San Diego, San Diego, California, USA; 2 Division of Biomedical Informatics, Department of Medicine, University of California San Diego, San Diego, California, USA

## 1 | INTRODUCTION

Intrahepatic cholestasis of pregnancy (ICP) is the most common hepatobiliary disease of pregnancy, and it is associated with spontaneous and iatrogenic preterm birth, meconium-stained amniotic fluid, neonatal intensive care unit (ICU) admission, and stillbirth [1–3]. It is postulated that elevated bile acids within fetal circulation cause cardiac myotoxicity and sudden stillbirth. Currently, there are no effective preventative or therapeutic interventions for ICP-associated stillbirth, except risk-reducing preterm to early-term delivery [1, 4].

In current practice, a single elevated maternal total bile acid (TBA) level is diagnostic for ICP and dictates the severity: mild (TBA 10–39 μmol/L), moderate (TBA 40–99 μmol/L), and severe (TBA ≥ 100 μmol/L). Given that adverse outcomes increase with increasing TBA levels, it appears reasonable to stratify this risk and individualize delivery timing [1–3]. However, despite contemporary studies better characterizing these adverse outcomes, clinical management paradigms do not yet reflect these changes.

## 2 | CLINICAL DILEMMA

In this viewpoint, we challenge the existing paradigm for the management of ICP including the contemporary risk stratification schema by proposing more evidence-based diagnostic cutoffs for mild ICP. Prior work has consistently shown that stillbirth rates with mild and moderate ICP are similar to the stillbirth rates in the general population, while higher rates are observed in cases of severe ICP [1, 2]. Similar trends are appreciated when assessing other ICP-associated adverse outcomes [2, 3].

In a nationwide administrative data analysis, Estin et al. noted lower rates of stillbirths among birth hospitalizations complicated by ICP than those without [4]. Although the current guidelines for early iatrogenic delivery for ICP reduced stillbirth, it came at a cost—higher rates of preterm birth among those with ICP (30.1% vs. 9.3%, *p* < 0.01) [4]. While the authors could not provide granular data on ICP severity, given the relative rarity of severe ICP (<1%), they proposed the possibility of unindicated iatrogenic preterm delivery for mild or moderate ICP cases contributing to this finding [4]. This suggests that our current ICP management may overcorrect and trade the perceived risk of stillbirth with the actual risks associated with preterm and early-term delivery. In other words, have we “solved” a problem that needs not be solved?

## 3 | STILLBIRTH RISK

**FIGURE 1:**
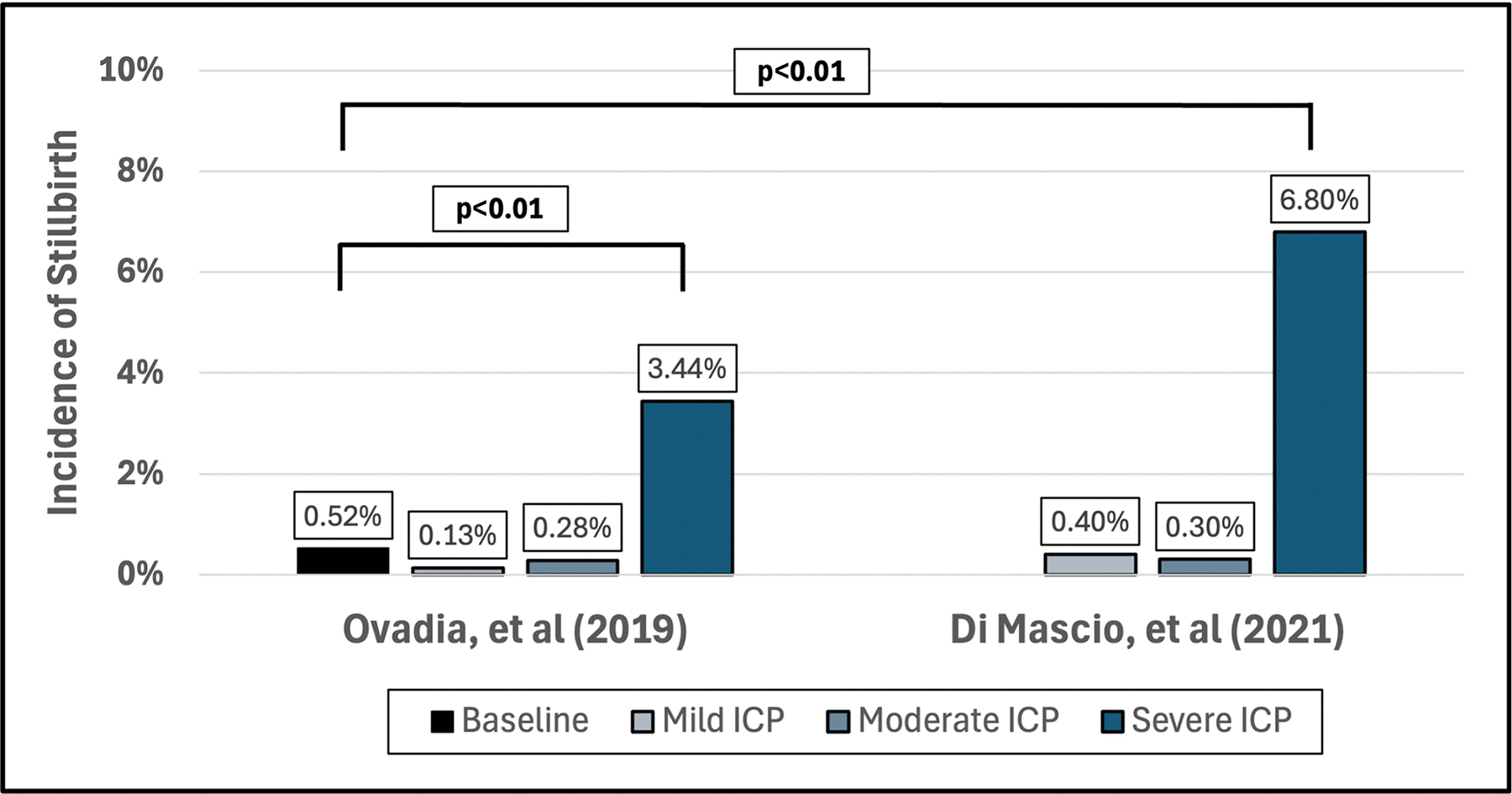
Incidence of stillbirth by ICP severity as reported by Ovadia et al. [1] and Di Mascio et al. [2]. Statistical comparison between studies was adapted and calculated independently for this clinical opinion. ICP, intrahepatic cholestasis of pregnancy.

In the last decade, there have been notable advancements in our understanding of ICP-associated stillbirth. Prior to 2019, risk profiles were poorly characterized, warranting iatrogenic early deliveries after balancing ICP-associated stillbirth and neonatal prematurity. In 2019, Ovadia et al. published a landmark study that characterized stillbirth risk by ICP severity [1]. Their study revealed that the incidence of stillbirth with ICP was 0.13% for mild, 0.28% for moderate, and 3.44% for severe (Figure 1) [1]. Notably, the stillbirth rates for mild and moderate ICP are not significantly different from those of the general population.

Subsequently, in 2021, Di Mascio et al. conducted a systematic review assessing ICP-associated stillbirth by ICP severity and found 0.4% for mild, 0.3% for moderate, and 6.8% for severe (Figure 1). Again, the stillbirth rates for mild and moderate ICP were similar to the baseline population risk, while significantly higher rates were seen among those with severe ICP only.

Direct comparison of stillbirth rates in meta-analyses poses a challenge as the general population encompasses risk across the entire gestation and the ICP cohort risk is typically confined to the third trimester. Despite this limitation, the estimates above highlight a significant worsening of stillbirth risk primarily with severe ICP.

## 4 | RISKS BEYOND STILLBIRTH

Di Mascio et al. also demonstrated an association between ICP severity and iatrogenic preterm birth (10.8% for mild, 21.6% for moderate, and 35.8% for severe) and meconium-stained amniotic fluid (9.0% for mild, 18.4% for moderate, and 31.6% for severe) [2]. Sarker et al., published a single center retrospective cohort study from 2005 to 2019 showing that increasing ICP severity was associated with increased rates of spontaneous preterm birth (4.8% mild, 10.7% moderate, and 19.2% severe), iatrogenic preterm birth (12.6% mild, 24.8% moderate, and 38.3% severe), and meconium-stained amniotic fluid (8.1% mild, 16.2% moderate, and 22.5% severe) [3]. Differences in stillbirth and other adverse outcome rates by ICP severity support reconsideration of delivery timing for mild ICP, permitting expectant management up to 39 weeks’ gestation.

## 5 | NEONATAL RISK OF IATROGENIC LATE PRETERM AND EARLY-TERM BIRTH

As noted above by Estin et al., there remains the possibility of unindicated iatrogenic preterm delivery for mild or moderate ICP cases in their nationwide sample. Both late preterm and early-term iatrogenic births are associated with numerous adverse outcomes. Neonates born in the late preterm and early-term periods are at an increased risk of respiratory distress syndrome, transient tachypnea of the newborn, ventilator use, pneumonia, respiratory failure, neonatal ICU admission, hypoglycemia, 5-min Apgar <7, and neonatal mortality [5]. Aside from the immediate adverse perinatal outcomes, studies have also reported long-term adverse consequences including increased hospitalizations up to age 18, slower neurologic development, worse cognitive performance, and poorer academic achievement [5]. While the question remains whether iatrogenic preterm birth for mild ICP prevents stillbirth, the risks of adverse outcomes with unindicated or unnecessary preterm birth are well established. The decision regarding the timing of delivery should weigh the risk of these adverse outcomes alongside the risks associated with pregnancy prolongation.

## 6 | CHALLENGING THE DIAGNOSTIC CRITERIA FOR MILD ICP

Given the lower risk of adverse outcomes with mild ICP, multiple studies and opinions have questioned the diagnostic cutoff. Notably, critics contend that mild ICP, specifically TBA levels of 10–19 μmol/L in the setting of pruritus, may simply be a diagnostic error resulting from laboratory or physiologic variation [6, 7]. Tamzali et al. assessed the variation of 11 clinically utilized TBA assays across seven laboratories in North America, Australia, and the United Kingdom and found that the normal reference ranges often included 10–19 μmol/L and, thus, were incongruent with the ICP diagnostic criteria of >10 μmol/L [7]. Additionally, Huri et al. conducted a cross-sectional study of 612 patients without clinical concerns for ICP to establish normal pregnancy reference ranges for TBA [6]. After assessing 528 fasting and 377 postprandial samples, they found that reference ranges include the diagnostic cutoff range for both fasting (4.4–14.1 μmol/L) and 2 h postprandial (4.7–20.2 μmol/L) [6].

These studies highlight the potential for incorrect diagnosis of normal variation as mild ICP, raising the question of whether the lower range of mild ICP ought to be included in the diagnostic criteria. However, we acknowledge that these studies remain limited in their small sample size and population diversity. Additionally, there may be gestational age–associated TBA variation that has not been accounted for in these studies.

## 7 | TIME TO DISCUSS REDEFINING THE MILD ICP DIAGNOSTIC CRITERIA AND CONSIDER A NEW MANAGEMENT PARADIGM

Contemporary outcome and laboratory assay studies provide robust support for a conversation on redefining diagnostic criteria and changing management for mild ICP. Given greater variation in benign TBA levels than previously considered, we feel that there is strong evidence to consider changing the diagnostic criteria for mild ICP from 10–39 μmol/L to 19–39 μmol/L (Table 1). While one study revealed that ursodeoxycholic acid therapy did not improve ICP-associated pregnancy outcomes [8], shared decision-making should determine initiation of therapy for symptomatic relief in patients with pruritus and TBA 10–18 μmol/L. However, these patients should not yet be diagnosed with ICP. Given that antenatal testing has not been shown to be beneficial for stillbirth prevention with ICP, patients with TBA 10–18 μmol/L also should not routinely be started on antenatal testing. However, as there remains the possibility of underdiagnosis due to physiologic or laboratory variation, we would recommend serial TBA testing in cases of TBA 10–18 μmol/L to follow trends with advancing gestation. Moreover, one study demonstrated that trending TBA values even after ICP diagnosis may more appropriately characterize ICP severity [9]. There is currently insufficient evidence to comment upon the timing of repeat serial TBA testing in patients with an existing diagnosis, and thus decision should be made on an individual basis with consideration for gestational age.

Historically, iatrogenic late preterm delivery for ICP seemed appropriate in the face of ill-defined and potentially catastrophic fetal risks. However, given the advancements in risk profiling and understanding of the implications of iatrogenic preterm or early-term delivery, a change in guidelines appears reasonable. Given similar stillbirth rates between patients with mild ICP and the general population, we feel early-term delivery for mild ICP is no longer warranted. Therefore, delivery prior to 39 weeks for mild ICP should not be recommended based on TBA levels alone (Table 1). If worsening maternal conditions are observed, such as liver compromise, delivery prior to 39 weeks for mild ICP may be justified. Quality of life considerations for symptomatic patients with mild ICP warrant shared decision-making for delivery timing.

We acknowledge that our proposed changes differ from the current Society for Maternal-Fetal Medicine (SMFM) guidelines [10]. However, these guidelines have been the standard of care for the Royal College of Obstetricians and Gynaecologists (RCOG) since 2022, the Society of Obstetric Medicine of Australia and New Zealand (SOMANZ) since 2023, and the Society of Obstetricians and Gynaecologists of Canada (SOGC) since 2024 [11–13]. All three of these international society guidelines have similar diagnostic criteria, with normal defined as TBA <19 μmol/L and mild ICP as TBA = 19–39 μmol/L. Additionally, in the absence of other stillbirth risk factors, they all have similar recommendations for planned delivery by ICP severity at the following gestational ages: mild 39–40 weeks, moderate 36–38 weeks (SOGC), and 38–39 weeks (RCOG and SOMANZ), and severe 35–36 weeks (Table 1) [11–13].

We suggest that contemporary evidence, aligned with guidelines from other international societies, supports changing the diagnostic criteria and delivery timing of mild ICP in the United States. However, given the possibility of baseline stillbirth differences between populations, our proposed delivery timing changes remain somewhat more conservative than international society guidelines. If implemented, the proposed changes will update SMFM guidelines to be consistent with those in practice by RCOG, SOMANZ, and SOGC.

## 8 | FUTURE AREAS OF STUDY

While our understanding and management of ICP have greatly evolved, there are areas that require further investigation. First, the testing interval, timing frequency, and clinical utility of TBA trending and how ursodeoxycholic acid therapy affects TBA trended levels warrant further study. Additionally, after ursodeoxycholic acid therapy, it remains unclear whether an improvement in TBA levels is associated with improved outcomes or whether the underlying disease process is ongoing. Second, a deeper understanding of TBA placental transfer threshold would allow for improved characterization of fetal risk based on TBA exposure severity. Third, examining fractionated bile acid profiles and adverse outcomes may help further risk-stratify patients. Finally, while delivery timing and pregnancy management are often guided by peak TBA, the role of worsening maternal pruritus or liver enzymes in the absence of worsening TBA warrants further investigation.

## Figures and Tables

**Table 1.** Proposed Changes to Diagnostic Criteria and Delivery Timing

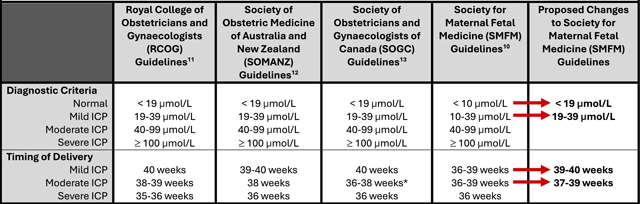

* Delivery timing by SOGC for moderate ICP further stratified to be 36-37 weeks for TB 40–69 μmol/L and 38 weeks for TBA 70–99 μmol/L13

## Data Availability

No original data were utilized for the formulation of this clinical opinion.

## References

[1] Ovadia C, Seed PT, Sklavounos A, Geenes V, Di Ilio C, Chambers J, Kohari K, 2019. “Association of Adverse Perinatal Outcomes of Intrahepatic Cholestasis of Pregnancy With Biochemical Markers: Results of Aggregate and Individual Patient Data Meta-Analyses.” Lancet 393: 899–909. 30773280 10.1016/S0140-6736(18)31877-4 PMC6396441

[2] Di Mascio D, Quist-Nelson J, Riegel M, George B, Saccone G, Brun R, Haslinger C, 2021. “Perinatal Death by Bile Acid Levels in Intrahepatic Cholestasis of Pregnancy: A Systematic Review.” Journal of Maternal-Fetal and Neonatal Medicine 34: 3614–22. 31744346 10.1080/14767058.2019.1685965

[3] Sarker M, Zamudio AR, DeBolt C, and Ferrara L. 2022. “Beyond Stillbirth: Association of Intrahepatic Cholestasis of Pregnancy Severity and Adverse Outcomes.” American Journal of Obstetrics and Gynecology 227: 517.e1–e7. 10.1016/j.ajog.2022.06.013 36008054

[4] Estin ML, Campbell AI, Watkins VY, Dotters-Katz SK, Brady CW, and Federspiel JJ. 2023. “Risk of Stillbirth in United States Patients With Diagnosed Intrahepatic Cholestasis of Pregnancy.” American Journal of Obstetrics and Gynecology 229: 453.e1–e8. 10.1016/j.ajog.2023.06.036 PMC10651178 37348778

[5] Borders AEB, Birsner ML, and Gyamfi-Bannerman C. 2019. “ACOG Committee Opinion No. 765: Avoidance of Nonmedically Indicated Early-Term Deliveries and Associated Neonatal Morbidities.” Obstetrics and Gynecology 133: e156–e63. 30681546 10.1097/AOG.0000000000003076

[6] Huri M, Seravalli V, Lippi C, Tofani L, Galli A, Petraglia F, and Di Tommaso M. 2022. “Intrahepatic Cholestasis of Pregnancy—Time to Redefine the Reference Range of Total Serum Bile Acids: A Cross-Sectional Study.” BJOG 129: 1887–96. 35373886 10.1111/1471-0528.17174 PMC9543426

[7] Tamzali I, Pirics ML, Bicocca M, and Burwick RM. 2022. “Reconsidering Absolute Diagnostic Thresholds in Intrahepatic Cholestasis of Pregnancy.” American Journal of Obstetrics and Gynecology 227: 784–6. 35839916 10.1016/j.ajog.2022.06.061

[8] Chappell LC, Bell JL, Smith A, Linsell L, Juszczak E, Dixon PH, Chambers J, 2019. “Ursodeoxycholic Acid Versus Placebo in Women With Intrahepatic Cholestasis of Pregnancy (PITCHES): A Randomised Controlled Trial.” Lancet 394: 849–60. 31378395 10.1016/S0140-6736(19)31270-X PMC6739598

[9] Sarker MR, Ramos GA, Ferrara L, and Debolt CA.2025. “Serial Total Bile Acid Measurements in Intrahepatic Cholestasis of Pregnancy.” Obstetrics and Gynecology 145(3): 343–5. 10.1097/AOG.0000000000005846. 39883945 PMC12077614

[10] Lee RH, Greenberg M, Metz TD, and Pettker CM. 2021. “Society for Maternal-Fetal Medicine Consult Series #53: Intrahepatic Cholestasis of Pregnancy.” American Journal of Obstetrics and Gynecology 224: B2–B9. 10.1016/j.ajog.2020.11.002 33197417

[11] Girling J, Knight CL, Chappell L, and Royal College of Obstetricians and Gynaecologists. 2022. “Intrahepatic Cholestasis of Pregnancy: Green-top Guideline No. 43 June 2022.” BJOG 129: e95–e114. 35942656 10.1111/1471-0528.17206

[12] Hague WM, Briley A, Callaway L, Dekker Nitert M, Gehlert J, Graham D, Grzeskowiak L, 2023. “Intrahepatic Cholestasis of Pregnancy—Diagnosis and Management: A Consensus Statement of the Society of Obstetric Medicine of Australia and New Zealand (SOMANZ): Executive Summary.” Australian and New Zealand Journal of Obstetrics and Gynaecology 63: 656–65. 37431680 10.1111/ajo.13719

[13] Hobson SR, Cohen ER, Gandhi S, Jain V, Niles KM, Roy-Lacroix M, and Wo BL. 2024. “Guideline No. 452: Diagnosis and Management of Intrahepatic Cholestasis of Pregnancy.” Journal of Obstetrics and Gynaecology Canada 46: 102618. 39089469 10.1016/j.jogc.2024.102618

